# Supplementary figures and images for: High-throughput saturation mutagenesis generates a high-affinity antibody against SARS-CoV-2 variants using protein surface display assay on a human cell
Source: PLoS Pathog. 2023 Feb 1;19(2):e1011119. doi: 10.1371/journal.ppat.1011119 (PMC9891525; doi:10.1371/journal.ppat.1011119)

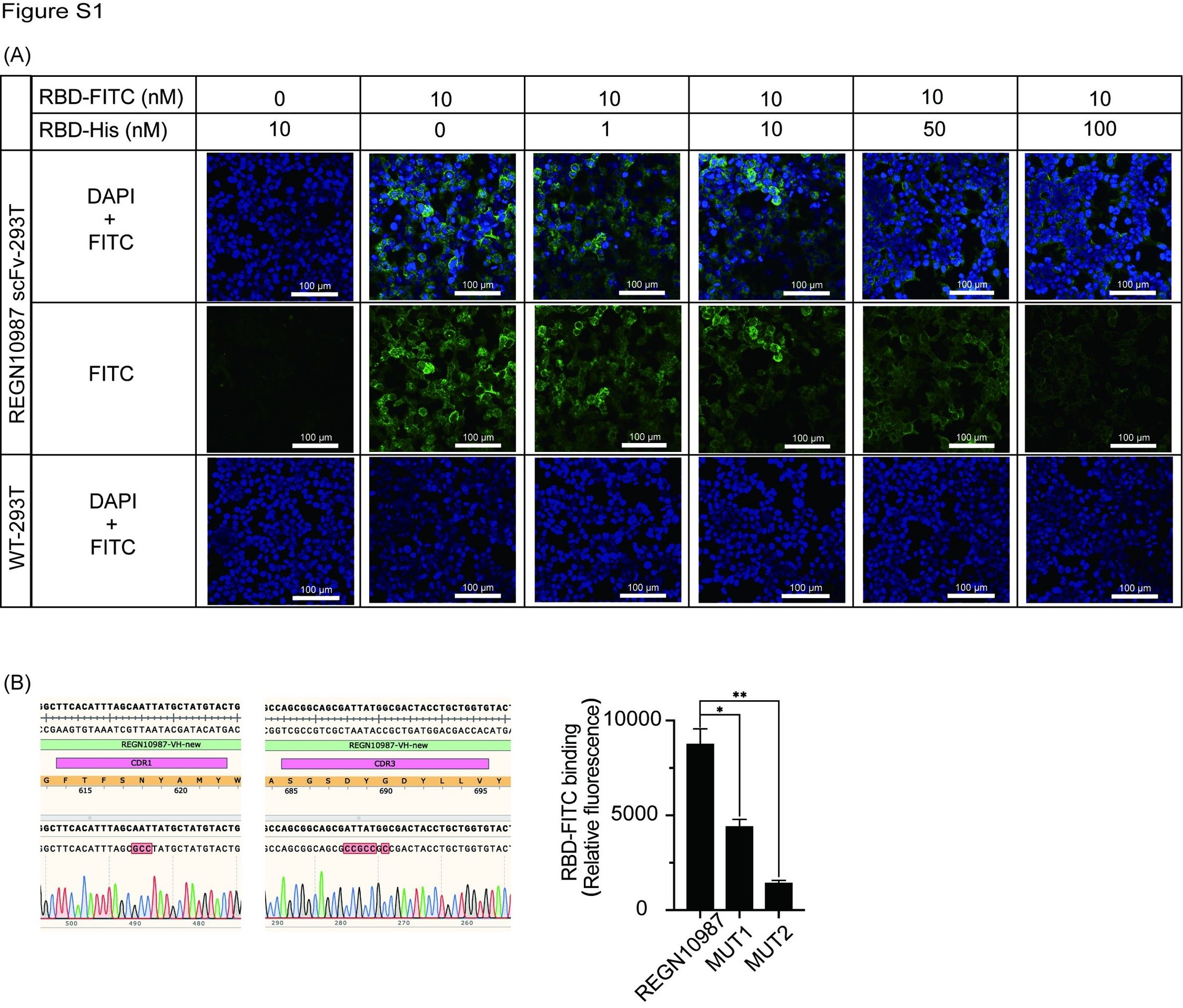

Supplement: S1 Fig — (A) Confocal microscopic images of RBD-His added to RBD-FITC competitively bound to REGN10987 scFv-expressing HEK 293T cells. Upper: HEK 293T cells expressed REGN10987 scFv. Lower: WT HEK 293T cells. Concentration of RBD-FITC was 10nM, while different titers of RBD-His was from 0 nM to 100 nM. Transfected cells were fixed with DAPI nuclear staining followed by detection with biotinylated RBD, followed by Streptavidin-FITC (green). Scale bar: 100 μm. (B) Four amino acids of key sites mutated into alanine in CDRs. Left: Sanger sequencing showed amino acid mutations, one sites in HCDR1 and three sites in HCDR3. Right: FITC signal for REGN10987 scFv and mutated scFvs displayed on HEK 293T cells binding with RBD-FITC after mutated key sites into alanine in CDRs. MUT1 contained three mutated amino acids in HCDR3, while MUT2 contained all of four mutated amino acids. Unpaired t test was used to analyze differences between groups. * p < 0.05, ** p < 0.01. (TIF) [file ppat.1011119.s001.tif]

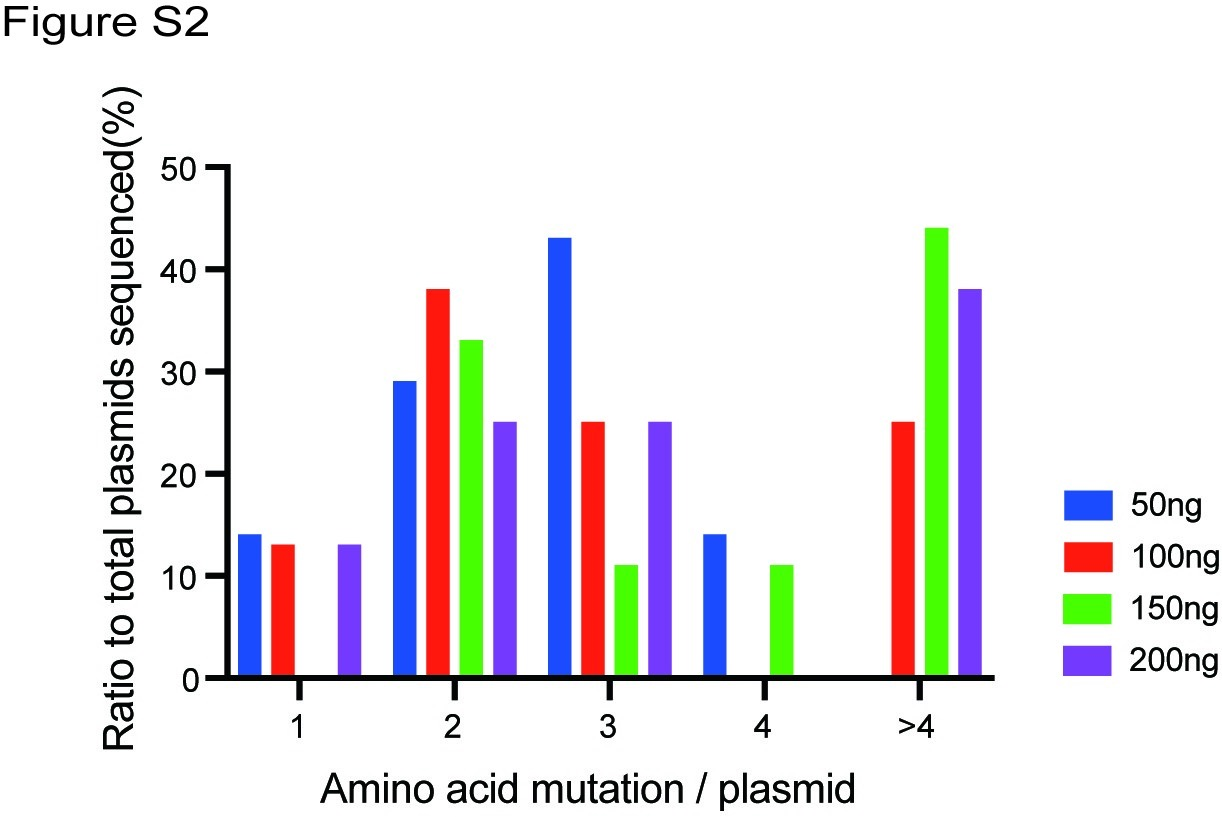

Supplement: S2 Fig — Ratios of amino acid mutations on REGN10987 scFv per plasmid based on a Sanger sequencing result of 32 pLV03 REGN10987 scFv mutation library plasmids. Concentration gradient of mutation primers from 50 ng/μL to 200 ng/μL was added in a PCR system. (TIF) [file ppat.1011119.s002.tif]

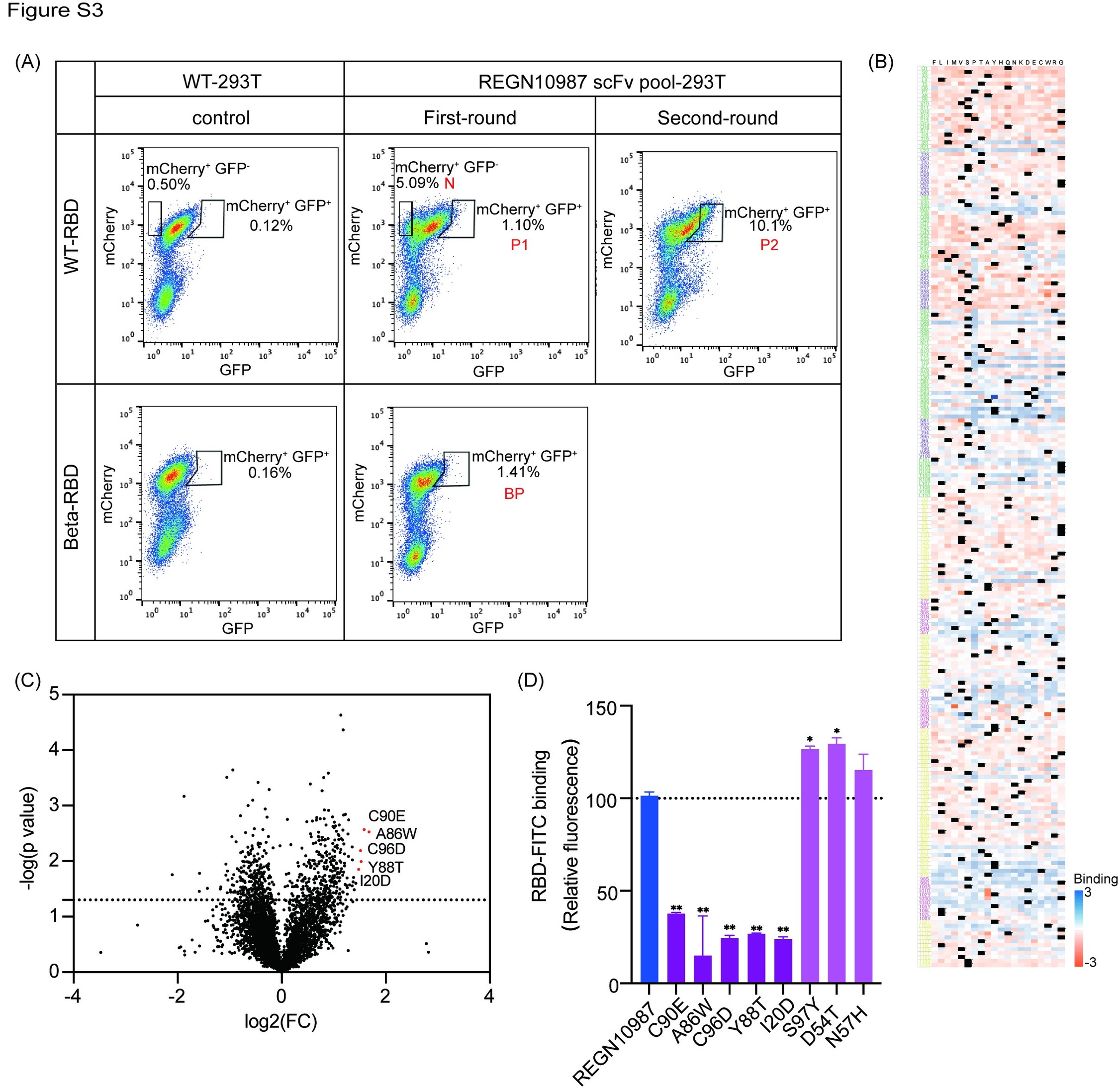

Supplement: S3 Fig — (A) FACS imaging for the cell mutant libraries in different screening rounds of WT-RBD (upper) and Beta-RBD (lower). P0 referred to the original cell library before screening. P1 and N referred to FITC-positive and FITC-negative cells respectively in the first round of screening by WT-RBD, and P2 referred to FITC-positive cells in the second round. BP referred to FITC-positive cells in the first round of screening by Beta-RBD. (B) REGN10987 scFv saturated mutagenesis heatmap of non-affinity binding to WT-RBD. Fitness scores based on the average Log2 enrichment ratios from two replications of the WT-RBD (N/P0) sorts were plotted from depletion or deleterious (orange) to enriched (blue). Positions on REGN10987 scFv were shown on the horizontal-vertical axis, and amino acid substitutions were indicated on the vertical axis. VL and VH of REGN10987 were shown in light green and yellow, respectively. CDRs of VL were shown in purple and CDRs of VH were shown in pink. (C) Volcano plots of increased and decreased amino acid mutations in binding affinity to WT-RBD of N groups. Representative mutations were highlighted in red. FC: relative fold change (mean frequency (after screening)/mean frequency (before screening)). Data were generated from n = 2 independent experiments. The p-value was calculated using a two-sided Student’s t-test. (D) Verification of single amino acid substitution REGN10987 scFv to WT-RBD using HEK 293T antibody surface display system. Data were normalized by REGN10987 scFv RBD binding fluorescence. Data are mean ± SEM, n = 2 replicates. Unpaired t test was used to analyze differences between groups. * p < 0.05, ** p < 0.01. (TIF) [file ppat.1011119.s003.tif]

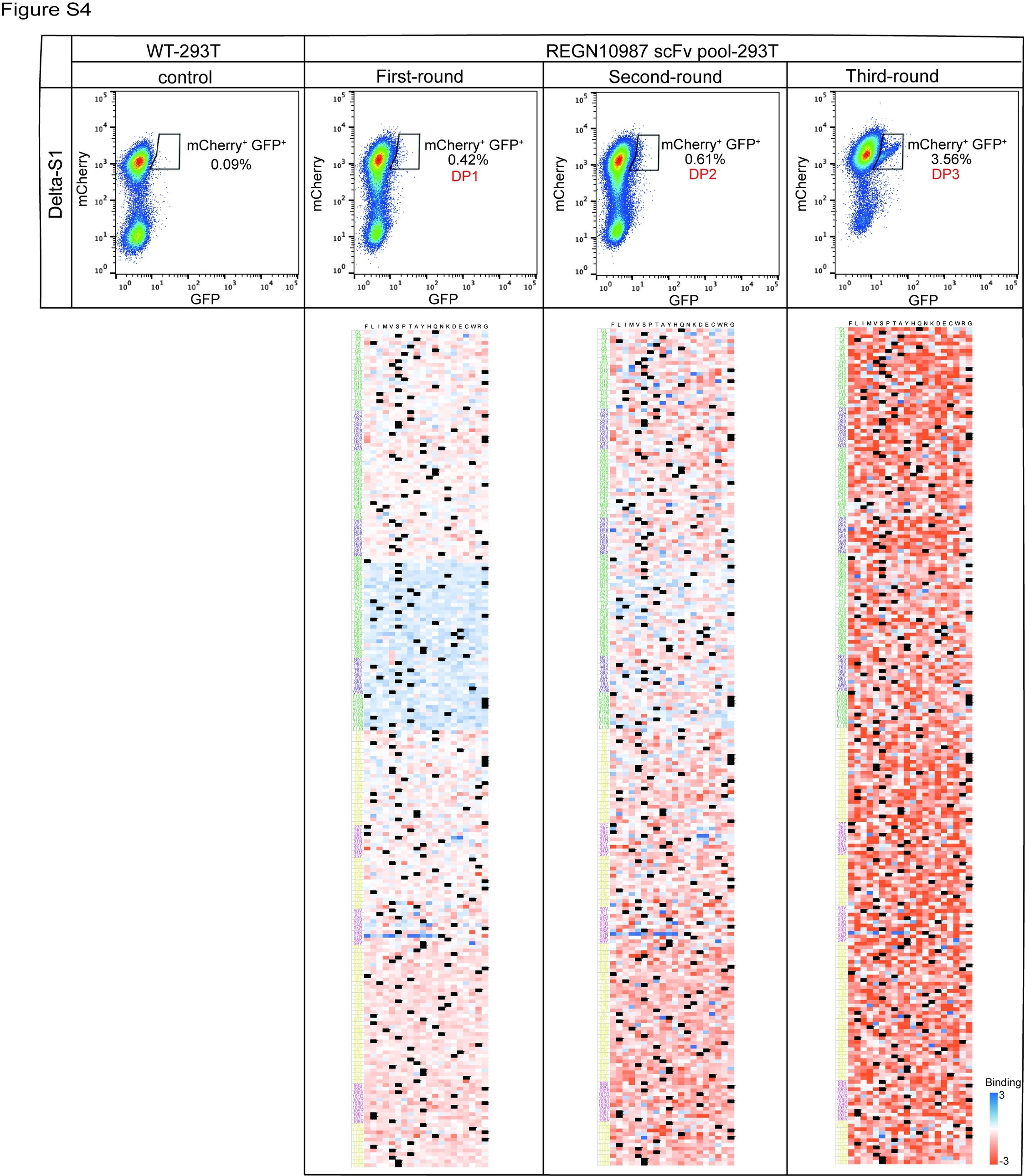

Supplement: S4 Fig — Upper: FACS imaging for the cell mutant libraries in three screening rounds of Delta-S1. DP1, DP2 and DP3 referred to FITC-positive cells respectively in the three rounds of screening. Lower: REGN10987 scFv saturated mutagenesis heatmap of high affinity binding to Delta-S1 in three screening rounds. Fitness scores based on the average Log2 enrichment ratios from two replications of Delta-S1 (DP1/DP0, DP2/DP0, DP3/DP0, respectively) sorts were plotted from depletion or deleterious (orange) to enriched (blue). Positions on REGN10987 scFv protein were shown on the horizontal-vertical axis, and amino acid substitutions were indicated on the vertical axis. VL and VH of REGN10987 were shown in light green and yellow, respectively. CDRs of VL were shown in purple and CDRs of VH were shown in pink. (TIF) [file ppat.1011119.s004.tif]

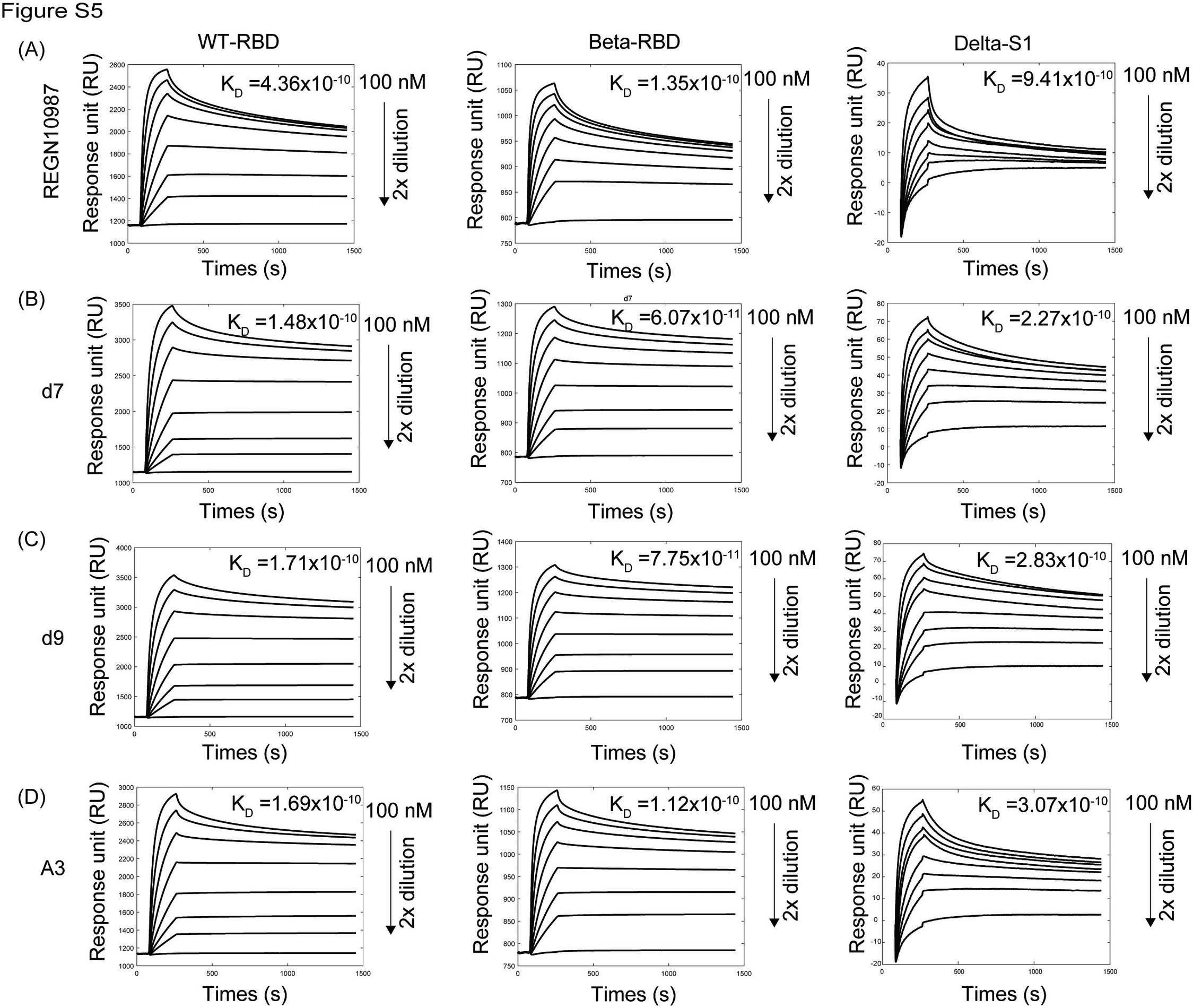

Supplement: S5 Fig — Immobilized WT-RBD, Beta-RBD or Delta-S1 association (t = 0 to 180 s) and dissociation (t > 180 s) with REGN10987 (A) and optimized antibodies d7 (B), d9 (C), A3 (D) measured by surface plasmon resonance (SPR). (TIF) [file ppat.1011119.s005.tif]

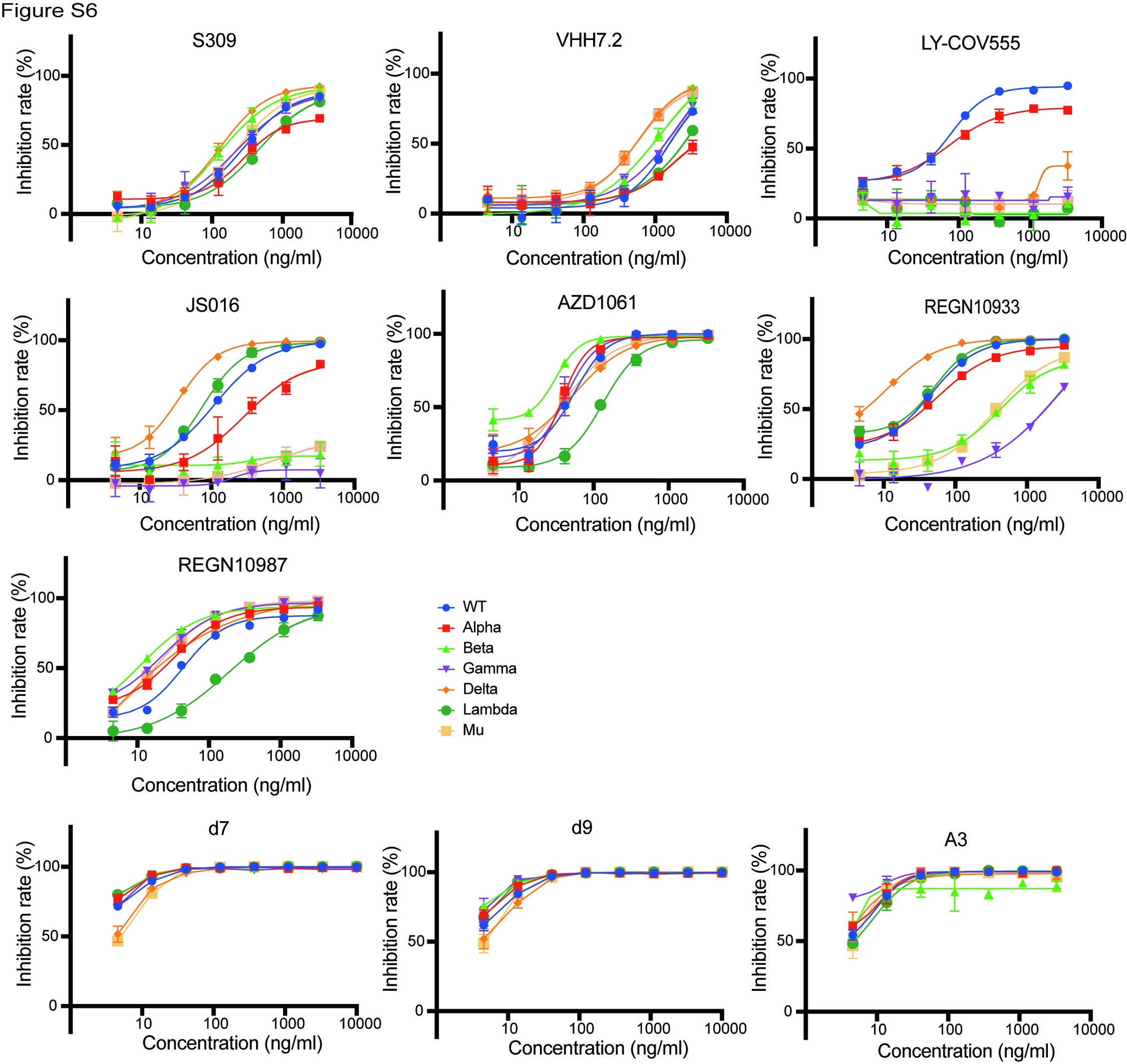

Supplement: S6 Fig — (TIF) [file ppat.1011119.s006.tif]
