# Supplementary material for: High-throughput saturation mutagenesis generates a high-affinity antibody against SARS-CoV-2 variants using protein surface display assay on a human cell
Source: PLoS Pathog. 2023 Feb 1;19(2):e1011119. doi: 10.1371/journal.ppat.1011119 (PMC9891525; doi:10.1371/journal.ppat.1011119)
Supplement: Attachment — Submitted filename: Response to Reviewer Comments.docx [file ppat.1011119.s007.docx]

We sincerely thank the reviewers for thoroughly examining our manuscript and providing very helpful comments to guide our revision. The responses to the comments are given below.

**Part I - Summary**
Please use this section to discuss strengths/weaknesses of study, novelty/significance, general execution and scholarship.

Reviewer #1: Review report for “High-throughput saturation mutagenesis generates a high-affinity antibody against SARS-CoV-2 variants using protein surface isplay assay on a human cell” by Yang et al submitted to PLoS Pathogens.

This study by JZ’s group and collaborators reports a high-throughput approach for generating high-affinity antibody against SARS-CoV2 variants. The strategy incorporates saturation mutagenesis, microarray-based oligonucleotide synthesis, and single-cell screening assay to generate a group of large set of antibodies against diverse prevalent SARS-COV-2 variants based on the human antibody REGN10987. The process can be done within 2 weeks and improve the affinity of antibodies with EC50 below 5 ng/ml.
Overall, this is a very interesting and timely article. As most countries have removed the pandemic regulations, the incidence rate of COVID-19 has been sky-high. Even though the hospitalization and severe disease rates are not as high in the current variants that are circulating. The actual amount remain alarming, especially for regions that have a low vaccination. The development of potent neutralizing antibodies is critically important in the coming time. Technologically, the approach sounds and presents a systems strategy for targeting the ever evolving COVID variants. The short turnaround and significant enhancement in affinity for broad variants are very impressive. The writing is overall good, and the data are of high quality. I recommend rapid acceptance of the manuscript and notify WHO for this new development for addressing the pandemic.

Response: Thank the reviewer for the valuable comments and suggestions.

Reviewer #2: The authors have described in detail a novel antibody optimization approach to obtain high-affinity antibodies against SARS-CoV-2. After reviewing the manuscript, I found that brief description of the structure of the virus and the properties of the spike (S) glycoprotein would be useful to aid in understanding of the topic, including on why the receptor binding domain (RBD) is often targeted. I would suggest including more details in the introduction on the other techniques used for antibody generation. For example, what are the authors referring when mentioning “the serious problems related to antibody activity” (pg 2, paragraph 2)?

Response: Thank the reviewer for the valuable comments and suggestions.

On page 2 paragraph 1, we have added “SARS-CoV-2 with trimeric spike (S) glycoprotein is found to infect human cells by binding to a receptor named angiotensin converting enzyme II (ACE2), and the interaction between S protein and ACE2 was emphasized to be a critical step for viral entry and infection [4, 5]”.

On page 3 paragraph 1, we added “Therefore, serious problems related to antibody activity, such as different glycosylation modifications that may dramatically change the features and functions of antibodies, can occur when proteins screened in bacterial or yeast systems are transferred to mammalian expression systems [33-36]”.

Please ensure that “SARS-CoV-2” is spelt with the appropriate case throughout the manuscript, including the abstract, i.e. SARS-CoV-2 and not SARS-COV-2.

Response: Thanks for your kind suggestions. We are very sorry for our typo in the abstract, and we have corrected them to SARS-CoV-2.

**Part II – Major Issues: Key Experiments Required for Acceptance**
Please use this section to detail the key new experiments or modifications of existing experiments that should be absolutely required to validate study conclusions.

Generally, there should be no more than 3 such required experiments or major modifications for a "Major Revision" recommendation. If more than 3 experiments are necessary to validate the study conclusions, then you are encouraged to recommend "Reject".

Reviewer #1: none

Reviewer #2: (No Response)

**Part III – Minor Issues: Editorial and Data Presentation Modifications**
Please use this section for editorial suggestions as well as relatively minor modifications of existing data that would enhance clarity.

Reviewer #1: Here are several optional suggestions to be considered by the authors.
1) The platform appears to be high versatile and may be applicable to address other emerging concerns, such as monkey pox and other infectious diseases. It will be useful if the author to expand the discussion to discuss the potential translation of the platform other diseases.

Response: Thank the reviewer for the constructive comments and suggestions. On page 16 paragraph 3, we have added “The same is true for mutated viruses that may appear in other species, such as monkey pox. In addition, this simple and efficient mammalian cell screening system can also be easily implemented to engineer other antibodies or proteins in the field of cancer immunotherapies”.

2) The use of feedback control and other AI based strategies has recently been adopted in the personalized medicine space. Are there efforts in using AI based approaches for optimizing potent antibodies? The author may consider discussing the compatibility between the platforms.

Response: Thanks to reviewer for value suggestion. It would definitely become more effective to combine artificial intelligence with our method for the screening of high affinity antibodies. On Page 16 paragraph 3, we added “Meanwhile, it would become more accurate and faster to screen high-affinity antibodies if our approach could be combined with feedback control and other AI based strategies”.

Reviewer #2: Suggested amendments/additions below are in parantheses:


Introduction
Page 2 Paragraph 1:
“So far, hundreds of millions of people have been confirmed infections and millions confirmed deaths (World Health Organization).” The wording here is confusing, the sentence should read “So far, there have been hundreds of millions confirmed infections and millions of confirmed deaths (World Health Organization).” Is there a specific WHO reference for this as well?

Response: Thanks to the reviewer for suggestion. We have made modification as suggested on Page 2 paragraph 1. We also added the specific reference for this description (reference 3 as shown in Page 22).

“As the epidemic progress, mutated strains with immune escape ability are emerging. The most serious strain is (the) Omicron strain that carries over 30 mutations on the S protein, half of which are located in the RBD. There is striking evidence that SARS-(CoV)-2 variants, particularly Omicron, have been less sensitive or completely escaped (from) antibodies [11-20].”

Ten references were cited here – suggested to elaborate on the antibody evasion of the SARS-CoV-2 variants mentioned. Which of the variants (Omicron, and others such as Delta) had especially reduced sensitivity to neutralising antibodies, and why?

Response: Thank the reviewer for the valuable comments. We made the correction. On page 2 paragraph 1, we also added the description and the explanation “As the epidemic progress, mutated strains with immune escape ability are emerging and have become less sensitive or completely escaped to antibodies. Five of those mutants were classified as variants of concern (VOC) due to their greater influence, namely Alpha, Beta, Gamma, Delta and Omicron. Some neutralizing antibodies, such as sotrovimab, were slightly less active against Alpha variant due to N501Y. The neutralizing activity of bamlanivimab and casirivimab against Beta variants was completely or significantly lost due to E484K and K417N mutations. The Gamma mutant escaped etesivimab by carrying K417T. Bamlanivimab lost its antiviral activity against the Delta variant that carries L452R. Strikingly, the majority of EUA-approved or advanced clinical development therapeutic neutralizing antibodies failed to neutralize the Omicron mutant due to the presence of 34 mutations, 15 of which are located in the RBD region [14-23]”.

Page 3 paragraph 1:
“…which was initially isolated from convalescent patients(’) B cells.”
Response: Thanks for reviewer’s kind suggestions. We made the correction.
Methods
Under the Flow Cytometry section: “To (bind) RBD to antibody REGN10987 scFv…”
Response: Thanks for reviewer’s kind suggestions. We made the correction.
Results
Page 6 paragraph 2:
Figure 2B was mentioned here but there appears to be a typo: “Fig. 2bB” should be “Fig. 2B”.
Response: Thanks for your kind suggestion. We have corrected it as “Fig. 2B”.

Page 6 paragraph 4:
“Finally, we (used) the next generation of DNA sequencing technology to decode…”
Response: Thanks for reviewer’s kind suggestions. We made the correction.
“All of (the) designed mutations (4,370) were observed in P0 group.”
Response: Thanks for reviewer’s kind suggestions. We made the correction.
“If needed, more (rounds) of selection could be performed.”
Response: Thanks for reviewer’s kind suggestions. We made the correction.
Page 9 paragraph 2:
“(In total), 1,541 amino acid mutations of WT RBD and 1,868 amino acid mutations…”
Response: Thanks for reviewer’s kind suggestions. We made the correction.
“To ensure that the binding affinity was (improved for) both WT and Delta SARS-CoV-2, an additional substitution mutation L106N was picked from the results of WT RBD, which was located in VH’s CDR3 that is believed (to be) an important recognition region for antigen recognition.”
Response: Thanks for reviewer’s kind suggestions. We made the correction.

Discussion
Page 15 paragraph 1:
“As the virus continues to evolve, more and more variants (will) appear in the infected population.”

What are examples of the variants of concern (VOC) mentioned here, and what are the mutating strategies that they employ for immune escape? Suggest to elaborate further.

Response: Thanks for reviewer’s kind suggestions. We made the correction. This question looks similar to the other question above, so we combined the two questions and added the description and the explanation “As the epidemic progress, mutated strains with immune escape ability are emerging and have become less sensitive or completely escaped to antibodies. Five of those mutants were classified as variants of concern (VOC) due to their greater influence, namely Alpha, Beta, Gamma, Delta and Omicron. Some neutralizing antibodies, such as sotrovimab, were slightly less active against Alpha variant due to N501Y. The neutralizing activity of bamlanivimab and casirivimab against Beta variants was completely or significantly lost due to E484K and K417N mutations. The Gamma mutant escaped etesivimab by carrying K417T. Bamlanivimab lost its antiviral activity against the Delta variant that carries L452R. Strikingly, the majority of EUA-approved or advanced clinical development therapeutic neutralizing antibodies failed to neutralize the Omicron mutant due to the presence of 34 mutations, 15 of which are located in the RBD region [14-23].” on page 2 paragraph 1.

Page 15 paragraph 3:
Authors mentioned “Recent advances in DNA synthesis techniques are expected to solve these problems well [40, 41]”. What advances in DNA synthesis techniques are the authors referring to?

Response: Thanks to reviewer for value suggestion. On page 15 paragraph 3, we added “Recent advances in DNA synthesis techniques, such as oligonucleotide microarrays that allow the non-degenerate saturation mutagenesis of contiguous codons, are expected to solve these problems well [43, 44]”.

With the use of various techniques, what is the feasibility of using the described mammalian-cell surface display system in lower resource settings? Perhaps this can be discussed as well.

Response: Thank the reviewer for the valuable comments and suggestions. On page 16 paragraph 3, we added “Generally, it takes much time and effort to separate and validate effective antibodies from the serum of recovered patients infected with a newly emerged mutant virus. Indeed, it is also difficult to obtain and handle patients’ serum without extra professional equipment and skill. Nevertheless, our mammalian cell surface display system can be operated in ordinary biological laboratories”.

Page 16 paragraph 1:
“This is probably because the change (in the) N57 site, included in all optimized antibodies, may play a key role in antigen recognition and binding.”

Response: Thanks for reviewer’s kind suggestions. We made the correction.
